
JOURNAL INFORMATION
==============================
NLM Title Abbreviation: Wirtschaftsdienst
Journal ID: Wirtschaftsdienst
NLM Journal ID: 101086612

Wirtschaftsdienst (Hamburg, Germany : 1949)

ISSN: 0043-6275

ARTICLE INFORMATION
==============================
PMCID: PMC8536916
PMID: 34720235
DOI: 10.1007/s10273-021-3033-z
Article ID: 3033
Article version: 1
Subjects: Ökonomische Trends

Konjunkturschlaglicht: Aufschwung am Arbeitsmarkt? Gemischte Signale

Economic headline: Upswing in the labour market? Mixed Signals

Wolf André wolf@hwwi.org

grid.435054.3 0000 0001 2227 8589 Hamburgisches WeltWirtschaftsInstitut (HWWI), Oberhafenstraße 1, 20097 Hamburg, Deutschland
Electronic publication date: 2021 Oct 23
Print publication date: 2021
Volume: 101
Issue: 10
First page: 831
Last page: 832
Copyright: © Der/die Autor:in 2021
License: Open Access: Dieser Artikel wird unter der Creative Commons Namensnennung 4.0 International Lizenz veröffentlicht (https://creativecommons.org/licenses/by/4.0/deed.de).
Open Access wird durch die ZBW — Leibniz-Informationszentrum Wirtschaft gefördert.
License URL: https://creativecommons.org/licenses/by/4.0/

issue-copyright-statement: © The Author(s) 2021

==============================

Am deutschen Arbeitsmarkt verdichten sich die Anzeichen für einen allgemeinen Aufschwung. Nach dem pandemiebedingten Anstieg der Arbeitslosigkeit zu Beginn des Jahres 2021 hat sich die im April einsetzende Erholungstendenz mittlerweile verstärkt. Im September 2021 ist die Arbeitslosenquote im Vergleich zum Vormonat von 5,6 % auf 5,4 % gesunken und liegt damit auch deutlich unter dem Niveau von September 2020 (6,2 %) (vgl. Abbildung 1). Schon im August 2021 war die absolute Zahl der Arbeitslosen entgegen dem saisonalen Einfluss zurückgegangen. Im September lag der Bestand bei 2,46 Mio., im Vergleich zur Situation zwölf Monate zuvor ein Rückgang um fast 400.000 Personen. Auch die Zahl der gemeldeten freien Stellen hat sich gegenüber dem vergangenen Jahr erhöht: von 590.000 auf 799.000. Damit ist das vor Beginn der Pandemie vorherrschende niedrige Niveau an Arbeitslosigkeit fast wieder erreicht.

Ein tieferer Blick in die Daten offenbart strukturelle Effekte hinter der allgemeinen Aufwärtsbewegung. Am meisten verbessert hat sich die Erwerbssituation solcher Gruppen, die zuvor am stärksten von einer Zunahme der Arbeitslosigkeit im Pandemieverlauf betroffen waren. So ist im Vergleich zu August 2020 die Arbeitslosigkeit bei Männern etwas stärker gesunken (von 6,5 % auf 5,5 %) als bei Frauen (von 5,8 % auf 5,2 %). Auch ergab sich ein deutlich massiverer Rückgang der Erwerbslosigkeit bei ausländischen (von 15,3 % auf 12,8 %) als bei deutschen Staatsangehörigen (von 5,0 % auf 4,3 %), wenngleich die Niveauunterschiede hier nach wie vor beträchtlich sind. Zudem sank in der jüngsten Altersgruppe der Erwerbstätigen (15-24 Jahre) die Arbeitslosenquote weit deutlicher (von 6,2 % auf 4,7 %) als im ältesten Segment der über 55-Jährigen (von 6,1 % auf 5,8 %) (Bundesagentur für Arbeit, 2021a). Die unterjährige Dynamik verlief dabei bei allen betrachteten Gruppen jeweils ähnlich, sodass es sich hier um Unterschiede in der Intensität und nicht im zeitlichen Einsetzen von Erholungseffekten zu handeln scheint.

Die beobachteten Diskrepanzen zwischen soziodemografischen Gruppen stehen zum Teil in Zusammenhang mit berufsspezifischen Erholungseffekten. So lassen sich für September 2021 zwar in nahezu sämtlichen Berufsgruppen[1] Rückgänge im Bestand an Arbeitslosen mit entsprechendem Zielberuf im Vergleich zu September 2020 feststellen. Das Ausmaß an Erholung war je nach Beruf jedoch unterschiedlich stark ausgeprägt. Abbildung 2 stellt die Entwicklung bei den durch besonders starke Rückgänge gekennzeichneten Berufsgruppen dar. In den Top 5 finden sich hier wenig überraschend drei Tätigkeitsfelder im Bereich personennaher Dienstleistungen (Verkauf, Büro/Sekretariat, Speisenzubereitung). Die Aufhebung oder Abschwächung pandemiebedingter Restriktionen in jüngster Zeit hat sich hier erwartungsgemäß positiv auf

1 Abgegrenzt als 3-Steller-Ebene der Klassifikation der Berufe (KldB) 2010.

Abbildung 1
**Entwicklung der Arbeitslosenquoten nach Personengruppen in Deutschland**

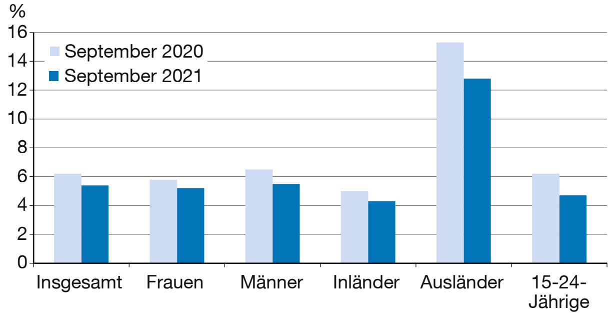

Quelle: Statistik der Arbeitsagentur (2021).

Abbildung 2
**Entwicklung der Arbeitslosenbestände nach Zielberufen in Deutschland**

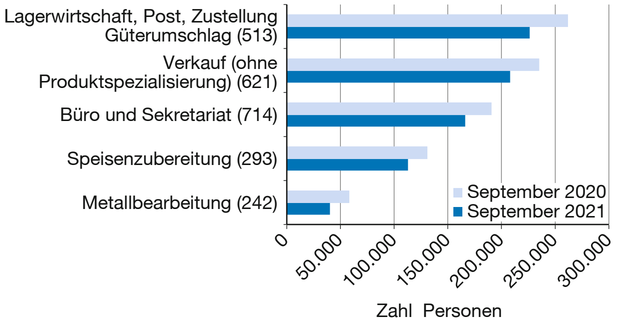

Quelle: Statistik der Arbeitsagentur (2021). In Klammern: Nummer KldB 2010 Statistik.

die Arbeitsnachfrage ausgewirkt. Dies zeigt sich jeweils auch in einer deutlichen Zunahme der Zahl an gemeldeten Arbeitsstellen. Die Stärke des Erholungseffekts spiegelt hier die Heftigkeit des Einbruchs im letzten Jahr: Alle fünf Berufsgruppen hatten im Zuge der Pandemie 2020 überdurchschnittlich starke Zunahmen im Arbeitslosenbestand erfahren. Ähnliches gilt für die ebenfalls stark von der Pandemie beeinträchtigten Gruppen „Gastronomie (633)“ sowie „Maschinenbau und -betriebstechnik (251)“. Allerdings zeigen sich bei anderen beschäftigungsseitig bedeutenden Berufsgruppen wie Objektschutz und Reinigungsberufen nur geringfügige Rückgänge im Arbeitslosenbestand. Diese Tätigkeitsbereiche waren weniger intensiv von der negativen Beschäftigungsentwicklung im Vorjahr betroffen (Bundesagentur für Arbeit, 2021b).

Damit gehen strukturelle Effekte einher. So waren 2020[2] in sämtlichen der sehr dynamischen Berufsgruppen mit Ausnahme von „Speisenzubereitung“ Frauen unter den Beschäftigten stark unterrepräsentiert, insbesondere im Bereich „Fahrzeugführung im Straßenverkehr“ (6,2 %) und „Maschinenbau und -betriebstechnik“ (12,1 %). Beschäftigte mit ausländischer Staatsangehörigkeit waren dagegen gegenüber ihrem Gesamtanteil (12,7 %) in den meisten der durch starke Erholungseffekte gekennzeichneten Berufsgruppen deutlich überproportional vertreten, vor allem in der „Speisenzubereitung“ (32,8 %) und der Lagerwirtschaft (26,0 %). Lediglich der Bereich „Büro und Sekretariat“ stellt hier eine Ausnahme dar (6,1 %) (Bundesagentur für Arbeit, 2020).

Dass die jüngere Entwicklung am Arbeitsmarkt relativ tätigkeitsspezifisch ist, zeigt sich auch am Muster der Abgänge aus der Arbeitslosigkeit. So haben von den zwischen Juli 2020 und Juni 2021 aus der Arbeitslosigkeit in reguläre Beschäftigung eintretenden Personen 18,5 % Verkehrs- und Logistikberufe ergriffen, ein gegenüber der Gesamtbeschäftigung in diesem Berufssegment stark überproportionaler Anteil. Auch der Einstieg in Lebensmittel- und Gastgewerbeberufe fiel mit 6,6 % überdurchschnittlich hoch aus. Hierbei zeigen sich charakteristische Unterschiede zwischen soziodemografischen Gruppen. So sind analog zur bisherigen Beschäftigungsstruktur Männer in deutlich höherem Maße als Frauen in Verkehrs- und Logistikberufe eingestiegen. Ebenso sind junge Erwerbstätige (15-24-Jährige) und Ausländer:innen unter den Neueingestellten überrepräsentiert. Unterschiede sind aber auch beim Anforderungsniveau der ergriffenen Beschäftigungen festzustellen. So ist hier der Anteil qualifikatorisch geringwertiger Helfertätigkeiten bei Männern deutlich höher als bei Frauen, auch bei Ausländer:innen deutlich höher als bei Inländer:innen. Dieser Struktureffekt sollte bei einer Beurteilung der Beschäftigungsperspektiven berücksichtigt werden (Bundesagentur für Arbeit, 2021c).

Zugleich erstreckt sich die insgesamt positive Erwerbsdynamik bislang nicht auf das Segment der Langzeitarbeitslosen. Die spezifische Langzeitarbeitslosenquote sank im September 2021 gegenüber dem Vormonat nur sehr geringfügig um 0,1 Prozentpunkte und liegt mit 2,2 % immer noch auf dem hohen Niveau des Jahresbeginns. Absolut ist die Zahl der Langzeitarbeitslosen gegenüber September 2020 sogar deutlich gestiegen (+17,2 %). Anteilig machen Langzeitarbeitslose damit gegenwärtig 41,8 % am gesamten Arbeitslosenbestand aus, ein Höchstwert innerhalb der vergangenen zehn Jahre. Bei der Zunahme handelt es sich ganz überwiegend um Übertritte aus kurzzeitiger Arbeitslosigkeit. Offenbar haben es unter den aktuellen Bedingungen vor allem die vor oder zu Beginn der Pandemie arbeitslos gewordenen Personen schwer, den Weg zurück in reguläre Beschäftigung zu finden. Auch dies gilt aber nicht im selben Maße für alle Gruppen. Auffällig ist vor allem der prozentual deutlich stärkere Anstieg der Langzeitarbeitslosigkeit unter Ausländer:innen (+27,8 % gegenüber September 2020) im Vergleich zu Inländer:innen (+13,3 %), was die insgesamt stärkere Verringerung der Arbeitslosenquote bei erstgenannter Gruppe etwas konterkariert (Bundesagentur für Arbeit, 2021d).

In näherer Zukunft wird es für einen umfassenden Aufschwung darauf ankommen, vorhandene Potenziale gerade bei den vom COVID-19-Schock besonders betroffenen Erwerbstätigengruppen zu reaktivieren. Förderangebote sollten dabei so weit wie möglich an die im Zuge der Pandemie veränderten Formen der Arbeitsorganisation angepasst werden, insbesondere im Hinblick auf die Schulung im Bereich digitaler Kompetenzen. Das Auftreten neuer gefährlicher Virusvarianten und damit zusammenhängender Beschränkungsmaßnahmen stellt hierbei naturgemäß ein exogenes Risiko dar.

André Wolf
wolf@hwwi.org

2 Gemessen zum Stichtag 30.6.2020.


Literatur

Bundesagentur für Arbeit (2021a), Statistik: Arbeitslosigkeit Im Zeitverlauf — Entwicklung der Arbeitslosenquote (Strukturmerkmale), September.
Bundesagentur für Arbeit (2021b), Statistik: Arbeitsmarkt nach Berufen (Monatszahlen), September.
Bundesagentur für Arbeit (2020): Statistik: Beschäftigte nach Berufen (KldB 2010), Dezember 2020.
Bundesagentur für Arbeit (2021c), Statistik: Abgang und Verbleib von Arbeitslosen in Beschäftigung, Juni.
Bundesagentur für Arbeit (2021d), Statistik: Langzeitarbeitslosigkeit (Monatszahlen), September.
